# Liposomes equipped with cell penetrating peptide BR2 enhances chemotherapeutic effects of cantharidin against hepatocellular carcinoma

**DOI:** 10.1080/10717544.2017.1340361

**Published:** 2017-06-23

**Authors:** Xue Zhang, Congcong Lin, Aiping Lu, Ge Lin, Huoji Chen, Qiang Liu, Zhijun Yang, Hongqi Zhang

**Affiliations:** a School of Chinese Medicine, Hong Kong Baptist University, Hong Kong, China;; b Changshu Research Institute, Hong Kong Baptist University, Changshu Economic and Technological Development (CETD) Zone, Changshu, Jiangsu Province, China;; c School of Biomedical Sciences, Chinese University of Hong Kong, Hong Kong, China;; d School of Traditional Chinese Medicine, Southern Medical University, Guangzhou, China

**Keywords:** Cell penetrating peptides, cancer cell specific peptide BR2, cantharidin, hepatocellular carcinoma, liposome drug delivery system

## Abstract

A main hurdle for the success of tumor-specific liposomes is their inability to penetrate tumors efficiently. In this study, we incorporated a cell-penetrating peptide BR2 onto the surface of a liposome loaded with the anticancer drug cantharidin (CTD) to create a system targeting hepatocellular carcinoma (HCC) cells more efficiently and effectively. The *in vitro* cytotoxicity assay comparing the loaded liposomes’ effects on hepatocellular cancer HepG2 and the control Miha cells showed that CTD-loaded liposomes had a stronger anticancer effect after BR2 modification. The cellular uptake results of HepG2 and Miha cells further confirmed the superior ability of BR2-modified liposomes to penetrate cancer cells. The colocalization study revealed that BR2-modified liposomes could enter tumor cells and subsequently release drugs. A higher efficiency of delivery by BR2 liposomes as compared to unmodified liposomes was evident by evaluation of the HepG2 tumor spheroids penetration and inhibition. The biodistribution studies and anticancer efficacy results *in vivo* showed the significant accumulation of BR2-modified liposomes into tumor sites and an enhanced tumor inhibition. In conclusion, BR2-modified liposomes improve the anticancer potency of drugs for HCC.

## Introduction

Currently, hepatocellular carcinoma (HCC) is difficult to treat clinically; effective targeted drug delivery systems are needed (Zhang et al., 2016c). Though liposomal delivery systems have been used with certain modification in a few clinical trials (Pattni et al., 2015), classical liposomes have been of little or no value as a carrier for anticancer drugs due to lack of specificity for given cancer cells and/or failure to penetrate tumor tissue (Liang et al., 2015). Many strategies have been deployed in order to improve penetration and help the carrier pass the cancer cell membrane. The use of cell-penetrating peptides (CPPs) is one promising approach to enhance cytosolic drug delivery (Walrant et al., 2013). CPPs are able to accelerate the absorption of macromolecules via physiological mechanisms such as energy-dependent endocytosis and energy-independent direct penetration (Zhang et al., 2016a). Despite these advantages, CPPs are not widely used due to lack of tissue-selectivity (Deshayes et al., 2005). If a way to deliver CPPs specifically to tumor tissue could be developed, it would greatly enhance their anticancer efficacy.

BR2, a 17-amino acid peptide with a sequence of RAGLQFPVGRLLRRLLR, is a derivative of the nonspecific cell-penetrating anticancer peptide buforin IIb (Jang et al., 2015). After stepwise elimination of the C-terminal regular α-helical motif RLLR, the novel peptide BR2 shows increased cancer cell specificity and hence minimized cytotoxicity to normal cells (Lim et al., 2013). In a previous study, BR2 showed about 4-fold higher efficiency of penetration into cancer cells than into normal cells compared with Tat, a well-known CPP (Koren and Torchilin, 2012), that has shown similar penetration efficiency regardless of cancer cell type (Torchilin and Levchenko, 2003; Lim et al., 2013). Possible reasons for why BR2 with two RLLR repeats displays cancer cell specificity include distinctive feature of cancer cells. The tumor cell membrane is characterized by a different membrane composition with altered fluidity, more negative surface charges, higher transmembrane potential and an increased level of acidic components on the surface compared to normal cells (Papo and Shai, 2003; Leuschner and Hansel, 2004). BR with one RLLR displays a weak cell-penetrating ability to cancerous cells, two RLLR repeats exhibit an efficient penetration into cancer cells without cytotoxicity to normal cells, whereas three RLLR repeats cause cell damage both in cancerous and normal cells. This unique feature of BR2 is of a great value that should be utilized as a CCP in liposomal delivery system for cancer treatment.

Cantharidin (CTD), the active compound isolated from the dried body of Chinese medicine blister beetles *(Mylabris phalerata or M. cichorii)*, has been used as an anticancer agent in traditional Chinese medicine for treatment of HCC and esophageal carcinoma for many years (Liu and Chen, 2009). CTD has been shown to be a strong and selective inhibitor of protein phosphatase 2 (Li et al., 1993), which plays a key role in the regulation of the cell cycle, apoptosis, growth, and cell-fate determination (Janssens and Goris, 2001). However, CTD is recognized as an extremely poisonous agent in clinic with a very narrow therapeutic window that hampers its use. In its ‘naked’ form, CTD is unable to reach solid tumor with high quantities without accumulating and harming healthy organs (van der Veldt et al., 2010). In addition, CTD used alone is reported to have poor solubility (0.029 g/l) thus low bioavailability (Oaks et al., 1960; Tenschert et al., 1987). These drawbacks of CTD could possibly be overcome if it is encapsulated in liposomes, a pharmaceutical carrier with the versatility of compatibility and biodegradability, particularly if the liposomes are further equipped with CPP for targeted drug delivery.

In this study, we postulated that BR2-modified liposomes loaded with CTD would target HCC cells with enhanced cytotoxicity and anticancer efficacy. To confirm the hypothesis and verify the aforesaid functions of the BR2 peptide, we designed a liposomal delivery system with biodegradable and biocompatible components, namely soybean lecithin and 1,2-Distearoyl-sn-glycero-3-phosphoethanolamine-*N*-[methoxy(polyethylene glycol)-2000] (ammonium salt) (DSPE-PEG_2000_). To confer the ability to specifically penetrate HCC, the BR2 peptide was firstly conjugated to the distal end of DSPE-PEG_2000_-maleimide. The BR2-modified liposomes were then prepared and characterized. *In vitro* cytotoxicity of BR2-Lp was examined by MTT assay on HCC cell line HepG2 and normal hepatocytes Miha cells. Cellular uptake and intracellular distribution studies were performed using coumarin 6 as a model hydrophobic fluorescent probe. The *in vitro* tumor penetration and its anticancer activity in a multilayer tumor spheroid were studied as well. Furthermore, the real-time biodistribution and *in vivo* anticancer efficacy of CTD encapsulated liposomes was performed in xenograft tumor model of Balb/c nude mice (Zhu et al., 2016).

## Materials and methods

### Materials and cells

Soybean lecithin (SPC) was purchased from Shanghai Tai Wei Chemical Company (Shanghai, China). DSPE-PEG_2000_ was bought from Avanti Polar Lipids (Alabaster, AL). DSPE-PEG-Mal (SUNBRIGHTDSPE-0.20MA) was purchased from NOF Co. Ltd. (Tokyo, Japan). BR2 peptide, cys-RAGLQFPVGRLLRRLLR, was provided by SciLight Biotechnology (Beijing, China). Cantharidin (CTD) was obtained from Chengdu Biopurify Phytochemicals Ltd (Sichuan, China). Coumarin 6 (cou6) and dimethyl sulfoxide (DMSO) were purchased from Sigma-Aldrich. Hoechst 33342 and the fluorescent LysoTracker Red DND-99 was purchased from Molecular Probes Inc. (Eugene, OR). Near-infrared (NIR) lipophilic carbocyanine dye 1,1′-dioctadecyltetramethyl indotricarbocyanine iodide (XenoLight DiR) was purchased from Caliper Life Sciences (Hopkinton, MA). 3-(4,5-Dimethylthiazol-2-yl)-2,5-Diphenyltetrazolium Bromide (MTT) was purchased from Invitrogen (Grand Island, NY). Other chemicals and reagents were of analytical grade.

Human cell line Miha (a non-tumorigenic, immortalized human hepatocyte cell line) (Pang et al., 2006) and HepG2 (ATCC, Manassas, VA) were maintained in Dulbecco's modified Eagle's medium (DMEM) supplemented with 10% fetal bovine serum (FBS) and 100 IU/mL penicillin, and 100 mg/mL streptomycin (Life Technologies, Carlsbad, CA), at 37 °C in a humidified incubator with a 5% CO_2_ atmosphere.

### Synthesis of DSPE-PEG2000-BR2

Cell-penetrating peptide BR2 with a sequence of RAGLQFPVGRLLRRLLR was used to investigate its penetrability in human HCC cells. It was conjugated to a modified lipid linker (DSPE-PEG_2000_-maleimide) before its conjugation to liposomes.

**Figure 1.**
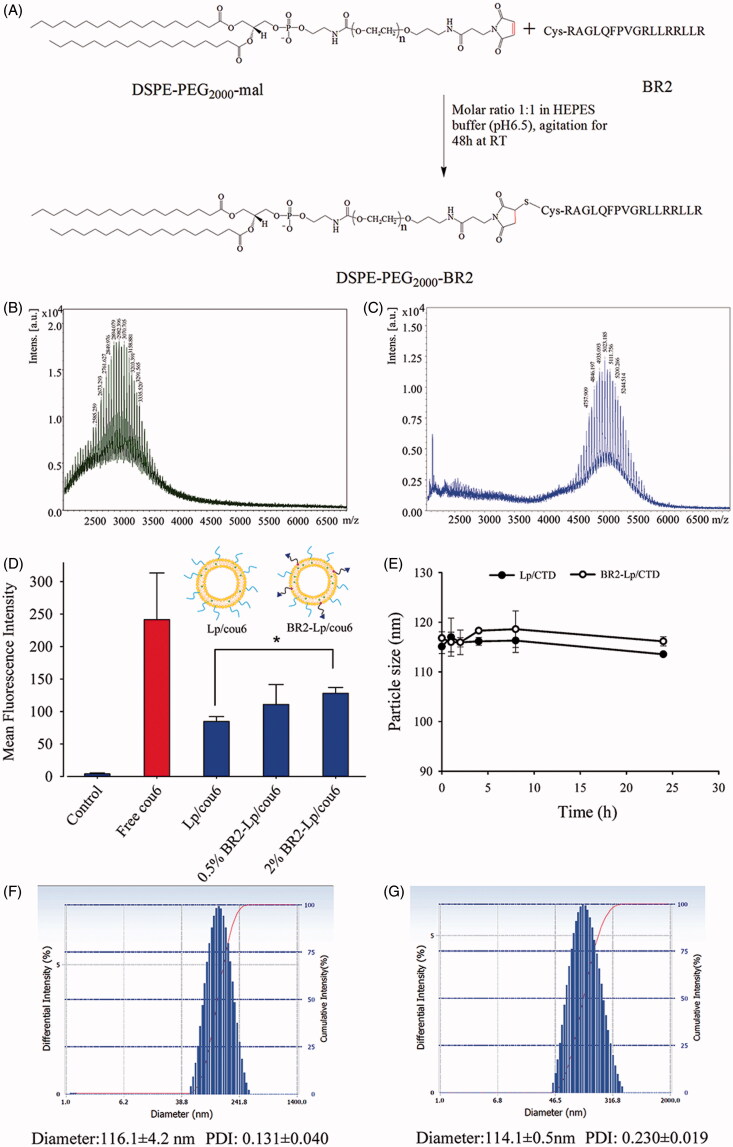
(A) Schematic diagram of conjugation of DSPE-PEG2000-BR2; (B) MALDI-TOF/TOF mass spectra of the DSPE-PEG2000-Mal and (C) MALDI-TOF/TOF mass spectra of the synthesized DSPE-PEG2000-BR2. (D) Effect of BR2 peptide on the cellular uptake of cou6-labeled BR2-modifed liposomes by HepG2 cells after incubation for 3 h at 37 °C. The auto-fluorescence of the cells was applied as control. Data presented as the mean ± SD, *n* = 3. *p < .05 when compared to Lp/cou6 group. (E) The variations in particle size of Lp/CTD and BR2-Lp/CTD follow the incubation time at 37 °C. The initial CTD concentration was 10 μM in different liposomes and equal volume of FBS was added. (F, G) Representative particle size distribution profile of Lp/CTD and BR2-Lp/CTD.

DSPE-PEG_2000_-BR2 was synthesized according to previous description (Yang et al., 2014) with slight modification (Figure 1(A)). Briefly, cysteine-modified BR2 (14.5 mg) was dissolved in 4 mL of HEPES buffer (20 mM HEPES, 10 mM EDTA-2Na, pH 6.5). Dried lipid film of 20 mg DSPE-PEG_2000_-Mal was hydrated in the HEPES buffer (1 ml) and added dropwise to BR2 peptide solution with gentle agitation at room temperature. After 48 h of stirring under nitrogen protection, the resulting solution was incubated with L-cysteine (10 times the molar ratio to maleimide residue) for another 4 h to react with any remaining maleimide group. The excess peptides and L-cysteine were removed by dialyzing the reaction mixture with a molecular weight cutoff (MWCO) of 3500 (Spectra/Por, Spectrum Labs, Rancho Dominguez, CA) against distilled water for 48 h. The solution was lyophilized and stored at −20 °C.

Successful formation of DSPE-PEG_2000_-BR2 was identified using a matrix-assisted laser desorption/ionization-time of flight (MALDI-TOF) mass spectrometer (Autoflex III; Bruker Daltonics Inc., Billerica, MA).

### Preparation and characterization of liposomes

Liposomes were prepared by ethanol injection method as described by Wong et al. (Wong et al., 2014). Lipid compositions of the prepared liposomes were as follows: (1) conventional PEGylated liposomes (Lp), SPC/DSPE-PEG_2000_ (molar ratio 96:4); (2) BR2-modified liposomes (BR2-Lp), SPC/DSPE-PEG_2000_/DSPE-PEG_2000_-BR2 (molar ratio 96:3.5:0.5 or 96:2:2). All lipids were dissolved in ethanol, and then hydrated with phosphate-buffered saline (PBS; pH 7.4) by rapid injection. Then the mixture was magnetically stirred for 1 h for liposome formation and ethanol removal. Afterward, the liposome suspensions were extruded through a 0.2 μm pore size filter one time and then through a 0.1 μm pore-sized filter (Millipore Co., Bedford, MA) five times. Cou6-labeled (Lp/cou6), DiR-labeled (Lp/DiR) and CTD-loaded (Lp/CTD) liposomes were prepared to form fluorescent-labeled and drug-loaded liposomes, respectively. Mean particle size and polydispersity index (PDI) of each formulation was assessed using the Delsa Nano HC Particle Analyzer (Beckman Coulter, Brea, CA). The CTD concentration in the liposome colloid solution was analyzed by gas chromatography-mass spectrometry (GC-MS) (Mehdinia et al., 2011). The percentage of CTD captured in liposomes was around 80%.

To investigate the effect of BR2 peptide density on cellular uptake, cou6-encapsulated BR2 peptide-modified liposomes (BR2-Lp/cou6) were prepared at different peptide densities (molar ratios of 0, 0.5 and 2%). HepG2 cells were seeded at a concentration of 5 × 10^5^ cells/well in six-well plates and incubated for 24 h. Then, the cells were incubated with different liposomal formulations at 0.1 μg/mL of cou6 for 3 h at 37 °C. After the incubation period, cells were trypsinized and washed three times with cold PBS. The cell suspensions were then centrifuged and resuspended with PBS and immediately subjected to analysis using a FACSCalibur flow cytometer (Becton Dickinson, San Jose, CA).

The serum stability characteristics of these two types of liposomes (Lp/CTD, BR2-Lp/CTD) with CTD concentration of 10 μM were investigated. The particle size was characterized as an indicator and the liposomes were co-incubated with 50% FBS based on literature (Zhang et al., 2016b). Briefly, liposomes were mixed with equal volume of FBS at 37 °C with moderate shaking at 30 rpm for 0, 1, 2, 4, 8 and 24 h incubation. Then 100 μl of the samples was pipetted out, diluted with 900 μl PBS, and the mixture was then transferred to a cuvette for particle size determination using a Delsa Nano HC Particle Analyzer.

### Cytotoxicity studies

The cytotoxicity of CTD-loaded Lp, BR2-Lp and free CTD against HepG2 and Miha cells was evaluated by MTT assay. HepG2 and Miha cells were seeded onto a 96-well plate at a density of 8 × 10^3^ cells per well. After 24 h incubation, the cell culture medium was replaced with fresh medium containing different formulations at CTD concentrations ranging from 10 to 100 μM, and cells were incubated for another 24 h. Then 20 μl MTT solution (5 mg/ml in PBS) was added to each well and cells were further incubated for 4 h at 37 °C. Then the medium was removed and 100 μl DMSO was added to each well to dissolve the formazan crystals formed by the living cells. Untreated cells in complete cell culture medium were used as control. The absorbance was measured by Benchmark Plus Microplate Reader at 570 nm. Cell viability (%) was calculated as *A*_treated_/*A*_control_ × 100%, where, *A*_treated_ and *A*_control_ represented the absorbance of cells treated with different formulations and blank culture medium, respectively.

### Cellular uptake of liposomes

Cellular uptake of Lp/cou6 and BR2-Lp/cou6 by both HepG2 and Miha cells was investigated by qualitative fluorescence imaging and quantitative flow cytometry. For fluorescent microscopy, 1 × 10^5^ cells per well were cultured in 24-well plates for 24 h followed by 3 h incubation with different liposomes at 0.1 μg/ml cou6 at 37 °C. The cells were then washed with cold PBS three times, fixed with 4% paraformaldehyde for 20 min and stained with Hoechst 33342 (5 μg/mL) for another 10 min. Finally, the cells were imaged and analyzed using a fluorescence microscope. For flow cytometry analysis, 5 × 10^5^ per well were seeded in a 6-well culture plate and cultured for 24 h for attachment. Then cells were incubated with cou6-loaded BR2-modified (BR2-Lp/cou6) and non-modified (Lp/cou6) liposomes for 3 h at 37 °C. For both, the final concentration of cou6 was 0.1 μg/ml. Probe-free culture medium was used as a blank control. After 3 h incubation, the culture medium containing liposomes was removed, and cells were washed at least 3 times with cold PBS. Then the cells were harvested using 0.5 ml 0.25% trypsin and centrifuged at 1000 rpm for 5 min. The supernatant was discarded, and pellets were re-suspended in 0.5 ml of PBS. The cellular uptake of fluorescence was analyzed on a flow cytometer equipped with laser (488 nm) and 530/30 band pass filters for emission measurements, and the data were analyzed using flowing software (version 2.5.1.; P. Terhu).

### Internalization of BR2-Lp in HepG2 cells

A fluorescent microscope was used to compare the intracellular distribution of the BR2-modified liposomes. Typically, 1 × 10^5^ of HepG2 cells/well were cultured on glass-bottom dishes containing complete medium for 24 h until total adhesion was achieved. Then, BR2-Lp/cou6 at 0.1 μg/ml cou6 were added to the culture medium and incubated at 37 °C for 0.5 h and 3 h. To track the internalization and endosomal release of liposomes, cells were washed and cultured with 100 nM LysoTracker Red in complete medium for 30 min at 37 °C to label endosome/lysosomes. The cells were then washed with cold PBS three times, fixed with 4% paraformaldehyde for 20 min and stained with Hoechst 33342 (5 μg/mL) for cell nuclei staining for another 10 min. Finally, the cells were imaged and analyzed using a fluorescence microscope.

### Tumor spheroid uptake and inhibition

#### HepG2 tumor spheroid development

HepG2 cellular three-dimensional (3 D) spheroids were established by hanging drop method as described (Zhang et al., 2013) with some modification. 1 × 10^3^ HepG2 cells in 200 μl complete culture medium were seeded onto a 96-well plate pre-coated with 80 μl 2% (w/v) agarose in FBS-free DMEM medium and incubated at 37 °C in the presence of 5% CO_2_. Spheroids were monitored under an inverted phase contrast microscope. The spheroids were ready to use after 7 days. Only uniform and compact tumor spheroids were selected for the follow-up studies.

#### Tumor spheroid penetration study

To detect the penetration properties of BR2-modified liposomes, *in vitro* 3 D tumor spheroids were developed as described above. Tumor spheroids were incubated with Lp/cou6, BR2-Lp/cou6 at a concentration of 0.5 μg/ml cou6 for 20 h and rinsed with cold PBS three times followed by fixing with 4% paraformaldehyde at room temperature for 20 min. Finally the spheroids were transferred to a chambered cover slip and the fluorescent intensity of different depths of tumor spheroids was observed with a confocal laser scanning microscope (Leica TCS SP8, Germany). The scanning of tumor spheroids proceeded from the top to the equatorial plane to obtain Z-stack images at 8 μm intervals. All images were taken using a 10x objective. Images were analyzed using Image-J software with Fiji package (NIH, version 1.51 d). To evaluate penetration profiles, cou6 intensity in the center of each Z-stack slice was calculated using corrected integrated pixel density as an indicator of the cou6 fluorescent intensity and plotted against the depth from top.

#### Tumor spheroid inhibition study

The growth inhibition of tumor spheroids was then investigated by analyzing relative spheroid volume changes and by MTT assay. Briefly, tumor spheroids of about 500 μm diameter were incubated with free CTD, Lp/CTD, BR2-Lp/CTD at a concentration of 10 μM CTD. The spheroids were then cultured for an additional 5 days. The spheroids were regularly imaged at 5x using an inverted phase microscope. The gray levels of tumor spheroids were processed by Image-J software with Fiji package to extract quantitative data (Schindelin et al., 2012). The length and width of each tumor spheroid were recorded and the volume was calculated with the formula: V = π×length × width^2^/6. The tumor spheroid volume change ratio was calculated as *R* =* V*_1_/*V*_0_×100%, where *V*_1_ is the tumor spheroid after treatment and *V*_0_ is the tumor spheroid volume before treatment (Liang et al., 2015). Volume curves were drawn to compare the effect of different treatments.

Cellular viability of tumor spheroids was determined after 5 days’ treatment of 7-day-old spheroids. After treatment with different formulations, six spheroids in each group were treated with MTT (5 mg/ml) and incubated for 4 h at 37 °C followed by dissolving with DMSO for 15 min. Then the cells were measured with Benchmark Plus Microplate Reader at 570 nm. The PBS-treated spheroid cells were taken as control to calculate cell viability.

### Animals and tumor model

Balb/c nude male mice (4-5 weeks, 18–20 g) were purchased from Laboratory Animal Services Center, The Chinese University of Hong Kong and acclimatized for 7 days after arrival. Nude mice were housed in individually ventilated cages (IVC cages) of isolated ventilation to avoid microbial contamination. All experimental procedures were done according to guidelines of the Committee on the Use of Human & Animal Subjects in Teaching & Research of Hong Kong Baptist University and the Health Department of the Hong Kong Special Administrative Region. Subcutaneous tumor model was established by inoculating approximately 5.0 × 10^6^ cells/100 μL HepG2 human HCC cells subcutaneously into the right flank of the nude mice (Zhu et al., 2016).

### *In vivo* real-time fluorescence imaging

DiR loaded liposomes were used to investigate the tumor targeting efficacy in the HepG2 tumor bearing mice. When tumor volume reached 150-200mm^3^, mice were injected with free DiR solution, DiR labeled liposome (Lp/DiR) and BR2-modified liposome (BR2-Lp/DiR) at a single dose of 2.0 mg DiR/kg body weight via tail vein, respectively. At predetermined post-administration time points, mice were anesthetized with intraperitoneal injection of 5% chloral hydrate at 100 μl/20 g dose. Fluorescence of injected DiR was visualized at lateral position using an IVIS Spectrum system (Caliper, Hopking, MA). At 48 h after injection, the mice were sacrificed by cervical dislocation. Tumors and major organs such as liver, kidneys, spleen, lung and heart were collected for *ex vivo* imaging. Gray-scale photographic images and fluorescent images of each sample were analyzed and overlaid using Living Image software (Caliper).

### *In vivo* anticancer efficacy

*In vivo* anticancer activity of the BR2-modified liposomes was evaluated in the subcutaneous HepG2 tumor-bearing nude mice. Tumor volume was measured by Vernier Calipers. When the tumor volume reached 50–100 mm^3^ (assigned as day 1), tumor-bearing mice were randomly divided into four groups (*n* = 5), the control that received saline, the free CTD, Lp/CTD and the BR2-Lp/CTD (0.35 mg/kg) via tail vein injection at a 3 day interval six times during the experiment. The body weight and tumor size were monitored every 3 days. The volume of the tumor was calculated using the equation: V = [length × width^2^]/2. Body weight change ratio (%) was calculated by the following formulate: [*W*_i_ − *W*_0_]/*W*_0_, where *W*_i_ was defined as the body weight at day i, W_0_ was defined as the body weight of the initial treatment at day 1.

### Statistics analysis

All data are shown as means ± SD unless specified otherwise. All statistical analyzes were performed using Sigma Plot version 11.0 (Systat Software, Inc., San Jose, CA). Statistical analyzes were performed using a two-tailed *t*-test or two-way ANOVA. A *p* < .05 was considered to be significant (denoted by *), and a *p* < .01 was considered as highly significant (denoted by **).

## Results

### Synthesis of DSPE-PEG_2000_-BR2

As shown in Figure 1(A), DSPE-PEG_2000_-BR2 was synthesized *via* the thiol-ene “click” reaction of maleimide group with the sulfhydryl group of the BR2 peptide (Xiang et al., 2013). First, MALDI-TOF-MS analysis showed that the peak of DSPE-PEG_2000_-Mal was right-shifted after conjugation (Figure 1(B and C)). Second, the major peak at 2982.396 (Figure 1(B)) showing mass charge ratios of DSPE-PEG_2000_-Mal and the peak at 5011.756 (Figure 1(C)) showing mass charge ratios of DSPE-PEG_2000_-BR2 verified that the mean Mw of DSPE-PEG_2000_-BR2 was approximately 5011, close to the theoretical calculated mean Mw (5108.496) of the corresponding product. These numbers indicate that the BR2 peptide was successfully conjugated to the DSPE-PEG_2000_-Mal group. In Figure 1(C) there is no peak between 2000 and 3000 mass charge ratios, indicating that most of the BR2 peptide was conjugated to the DSPE-PGE_2000_-maleimide group.

### Preparation and characterization of different liposomes

The average diameter for all liposomes was similar, in a range of 100–130 nm, and had good uniformity with the PDI less than 0.250 (Figure 1(F and G)). The liposome with such an uniformity has been reported to endow the liposomal delivery system for better therapeutic effect by utilizing the enhanced permeability and retention (EPR) *in vivo* (He et al., 2010). Compared with unmodified liposomes, BR2 modification had no significant change on particle size for CTD or fluorescent probe-loaded liposomes.

Surface modification such as the density of ligands of liposomes is a crucial factor greatly influencing their interaction with targeted cells. Therefore, when preparing BR2 peptide-modified liposomes, it was necessary to screen the basic BR2 ratio in liposome constructs for further experiments. In this study, liposomes with varying BR2 peptide density of 0, 0.5, and 2% were prepared and fluorescence intensity of these liposomes after the various BR2-modified liposomes treatment in HepG2 cells was analyzed. As shown in Figure 1(D), for HepG2 cells, as the BR2 density increased, cellular uptake was enhanced, such that, when the BR2 peptide density was 0.5%, there was no significant increase of uptake compared to Lp/cou6 group (*p* > .05), but a significant increase of uptake at 2% BR2 (*p* = .036) compared with Lp/cou6 group. Hence, the 2% BR2 molar ratio was selected in the following experiments.

The liposome preparation was co-incubated with 50% FBS, which mimics physiological conditions in order to assess its expected stability in living tissue. Particle size, as an indicator of liposomal stability, did not change over 24 h in the presence of serum (Figure 1(E)), showing that the liposomes did not aggregate and that PEGylation stabilized the liposomes.

### Cytotoxicity studies of liposomes

**Figure 2.**
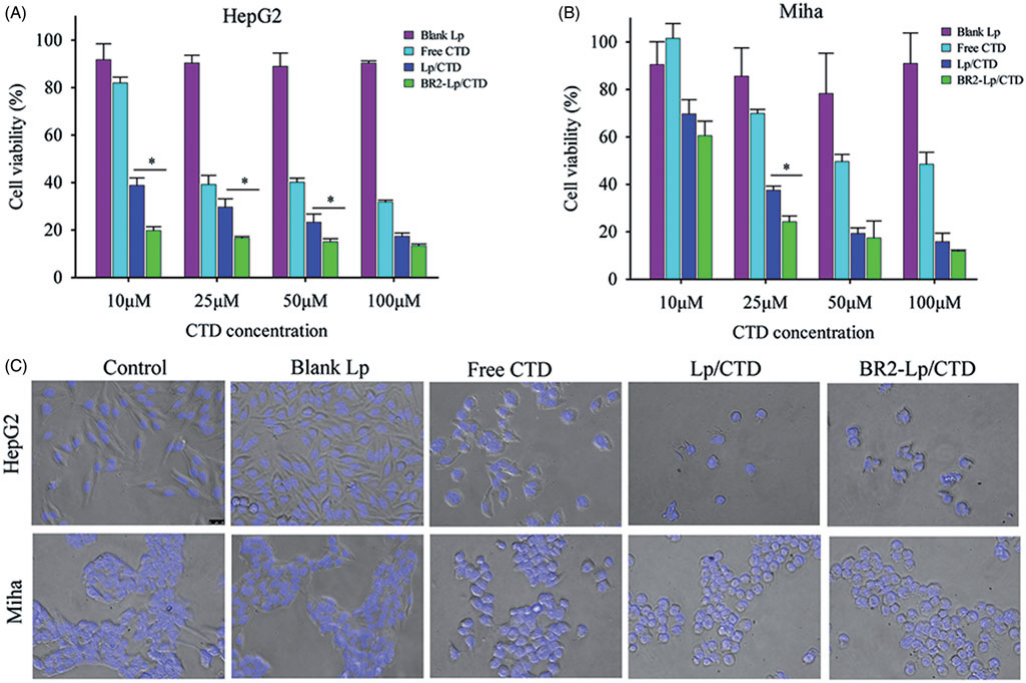
The cytotoxicity of various CTD formulations toward HepG2 (A) and normal hepatocytes Miha cells (B) by MTT assay. The cells were treated 24 h respectively with blank Lp, Lp/CTD, BR2-Lp/CTD and free CTD with indicated concentrations of CTD (10-100 μM). *p < .05 for BR2-Lp/CTD sample and Lp/CTD treatment groups. Lower panels (C) show the morphologies of HepG2 and Miha cells after incubated with CTD formulations at 10 μM for 24 h.

To evaluate the cytotoxicity of BR2-modified liposomes and to study the specific penetration of BR2 to cancerous cells instead of normal cells, HepG2 cells and the normal liver cells Miha were chosen. Free CTD, Lp/CTD and BR2-Lp/CTD were incubated with cells at CTD concentrations of 10–100 μM for 24 h, after which cell viability was evaluated by MTT assay. Compared to free CTD, Lp/CTD and BR2-Lp/CTD were more toxic to both HepG2 and Miha cells (Figure 2(A and B)). For HepG2 cells, BR2-Lp/CTD showed an enhanced cytotoxic activity compared with Lp/CTD. This difference can be attributed to BR2-modification, which improved the ability of liposomes to penetrate cells. Thus, the incorporation of the BR2 penetrating peptide played an important role in enhancing cytotoxicity. Indeed, BR2-Lp/CTD showed significantly higher cytotoxic effects on cancerous HepG2 cells than on normal Miha cells (Supplementary Appendix Figure A1), as expected.

In addition, we evaluated the cytotoxicity of blank liposomes to determine if liposomes themselves caused any cytotoxicity. The results indicated that blank liposomes had little toxic effect on either HepG2 or Miha cells. Hence, the enhanced cytotoxicity of BR2-Lp/CTD was most likely due to CTD internalized into cells.

The morphologies of the treated cells agreed with MTT results (Figure 2(C)). Most of the HepG2 cells attached and spread well on the cell culture plate in the control and the blank Lp groups, while after incubation with 10 μM CTD concentration for 24 h, all the cells detached and shrank to spheres in the Lp/CTD and BR2-Lp/CTD groups. Most importantly, BR2-Lp/CTD induced almost complete destruction of the cancer cells as evidenced by the presence of fragmented nuclei. The results indicated that the blank Lp had no cytotoxicity, while it was the BR2-Lp/CTD that destroyed HepG2.

### Different cellular uptake of BR2-Lp *in vitro*

**Figure 3.**
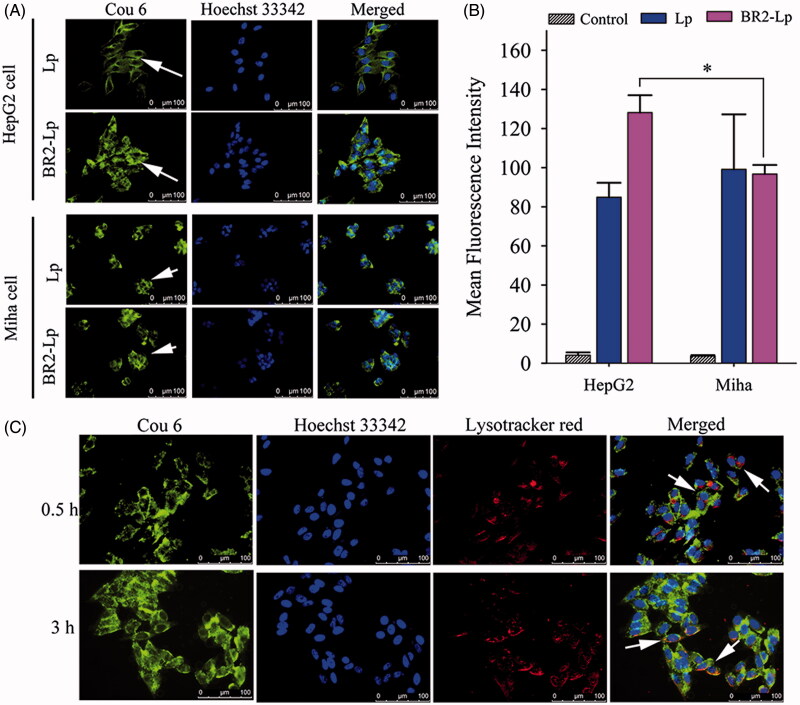
(A) Cellular uptake of coumarin-6 labeled Lp and BR2-Lp by HepG2 cells and normal hepatocytes Miha cells. Both cells were treated with Lp and BR2-Lp at 0.1 μg/ml cou6 for 3 h at 37 °C, respectively. Cell nuclei were stained blue with Hoechst 33342. Coumarin-6 was shown as green fluorescence (Scale bar 100 μm). The images were taken by fluorescent microscope. (B) Mean fluorescence intensity of cells after incubation with BR2-Lp/cou6 for 3 h at 37 °C by flow cytometry. The data presented as the mean ± SD, *n* = 3. *p < .05. (C) Intracellular distribution of cou6-loaded BR2-Lp at 0.1 μg/ml Cou6 concentration in HepG2 cells after 0.5 and 3 h incubation. BR2-Lp was represented in green fluorescence. Cell nuclei and endosomes/lysosomes were counterstained with Hoechst 33342 (blue) and LysoTracker Red (red), respectively. The scale bar represents 100 μm.

The cellular uptake of Lp/cou6 and BR2-Lp/cou6 in both HepG2 and Miha cells after 3 h incubation was determined either by fluorescent imaging or flow cytometry. From the qualitative results shown in Figure 3(A), liposomes were internalized into the cytoplasm and they were clustered around the nuclei in Lp treated HepG2 cells, where cou6 was detected with green fluorescence and the cellular nuclei were detected with blue fluorescence, having been stained with Hoechst 33342. This contrasted with BR2-Lp-treated HepG2 cells in which most liposomes had penetrated into the nuclei. However, Miha cells treated with Lp or BR2-Lp showed the liposomes had penetrated only into the cytoplasm. This result indicated that the BR2 peptide enabled liposomes to penetrate deep into tumor cells reaching the nuclei but not into the nuclei of normal cells.

As seen in Figure 3(B), BR2-Lp-treated HepG2 cells had stronger fluorescent intensity than that in Miha cells (*p* < .05), once again indicating that BR2-modified liposomes penetrated more deeply into HepG2 cancer cells than into normal cells.

### Cellular internalization of BR2-Lp in HepG2 cell

In order to demonstrate the cellular internalization of BR2-modified liposomes in HepG2 cells, the cou6-labeled BR2-Lp was imaged by fluorescent microscope after cells were incubated at 37 °C for 0.5 and 3 h. As shown in Figure 3(C), after 0.5 h, cou6-labeled BR2-Lp was mostly dispersed in the cytoplasm or localized around the nucleus. After 3 h, most liposomes were distributed around the cytoplasm and had even penetrated into the nuclei. Overall cellular uptake increased as the incubation time increased from 0.5 to 3 h.

To confirm co-localization with the lysosomes, the cellular acidic lysosome/endosome was stained with LysoTracker Red (Figure 3(C)). Although there was some overlap of lysosomes and liposomes (shown in yellow color, a combination of red and green fluorescence), most of the internalized BR2-Lp was present in the cytoplasm, but not in lysosome vesicles. This is probably because BR2 escaped lysosome entrapment; this reasoning is in line with a previous report (Lim et al., 2013).

### Tumor spheroid penetration

As seen in the image of HepG2 tumor spheroid growth in Supplementary Appendix Figure A2, tumor spheroids had been formed by day 4, and grew larger and denser with time. The tumor spheroids in day 7 were used in the following experiments.

**Figure 4.**
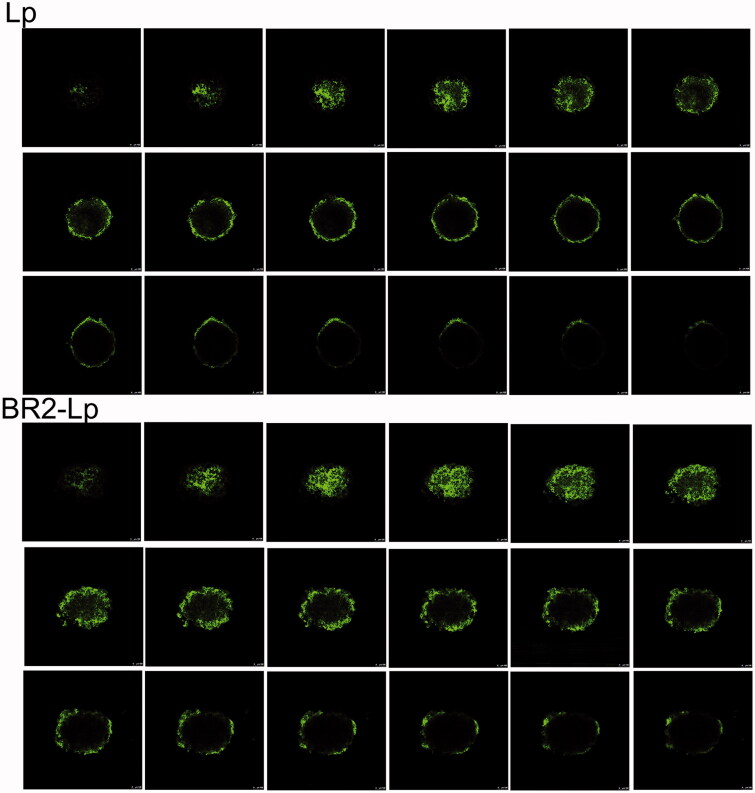
Confocal microscope images with an 8 μm interval between consecutive slides of HepG2 tumor spheroids after incubation with plain liposome and BR2-modified liposome with 0.5 μg/ml cou 6 concentration encapsulated for 20 h. The scale bar represents 100 μm.

The ability of different liposomes to penetrate HepG2 tumor spheroids was observed by confocal microscope. After incubation with liposomes, different layers of HepG2 tumor spheroids were scanned and imaged (Figure 4). In the unmodified liposome (Lp) group, fluorescence was mainly distributed in the core of the HepG2 spheroids from 64 μm to the top, with less found in the periphery. In contrast, in the BR2-modified liposome group, fluorescence was obviously distributed in the cores of HepG2 tumor spheroids from 88 μm to the top, and the fluorescence was still clear at 144 μm. Using the Z-stack plot, the fluorescence of each experimental liposome group was quantified as a function against spheroid depth (Supplementary Appendix Figure A3A). The profile clearly displays the increased penetration of BR2-Lp into the spheroids. In addition, the cumulative fluorescence from the spheroids was quantified from slice 8–144 μm (Supplementary Appendix Figure A3B). This clearly indicates that the conjugation with BR2 peptide enhanced liposomes ability to penetrate HepG2 tumor spheroids, while Lp without modification was unable to penetrate into the tumor spheroids.

### Tumor spheroids inhibition

**Figure 5.**
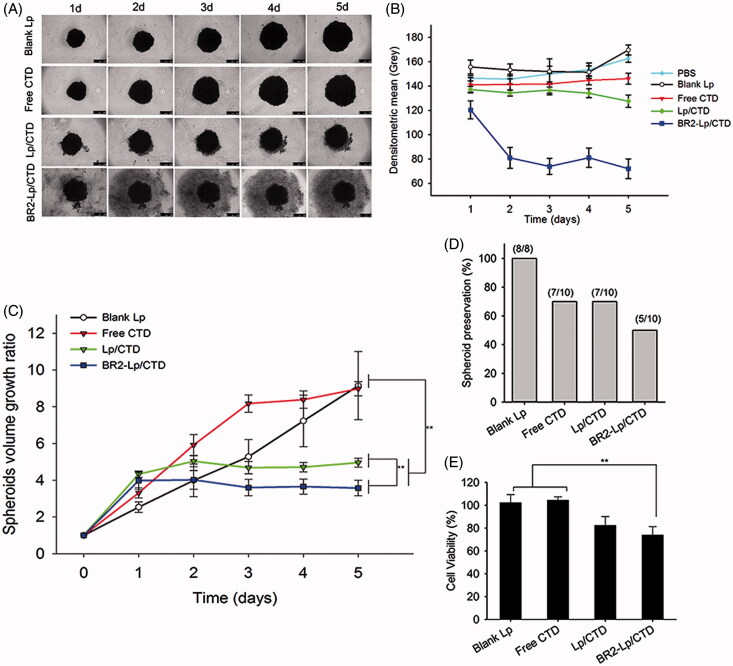
(A) Representative optical images of HepG2 tumor spheroids after exposure to a concentration of 10 μM CTD of different formulations. The scale bar represents 500 μm. (B) The mean gray levels of tumor spheroids in different groups were quantified by Image-J software with Fiji package. (C) Time-related volume of HepG2 tumor spheroids after treatment with different formulations at the same dose (10 μM CTD concentration) from 1 to 5 days. **p < .01. (D) HepG2 tumor spheroids preservation after 5-day treatment with different formulations. (E) Cell viability of the various formulations against HepG2 spheroids.

The cytotoxicity of blank Lp, free CTD, Lp/CTD, and BR2-Lp/CTD at 10 μM CTD concentration delivered to 3D tumor spheroids was then investigated using a growth inhibition assay (Figure 5). Figure 5(A) represents the tumor spheroid morphology changes after treatment with different formulations. Treatment of Lp/CTD and BR2-Lp/CTD both inhibited spheroid growth for 5 days as compared to blank Lp and free CTD group. In Lp/CTD and BR2-Lp/CTD groups, the tumor spheroids showed cells loosening and breaking up, especially on the perimeter, due to the cytotoxicity of CTD, especially in the BR2-Lp/CTD group. The morphology of spheroids showed cells very loosely attached to each other and, in comparison to the control spheroids, the well-defined spherical rim was lost as the incubation time increased. Figure 5(B) shows the gray levels of tumor spheroids in different groups. From this Figure it can be seen that, at day 5, the mean densities of tumor spheroids in the blank Lp group were slightly increased, while they were hardly changed in the free CTD group. In contrast, the mean density dropped dramatically in the BR2-Lp/CTD group which could be due to the cytotoxicity of BR2-Lp/CTD.

Figure 5(C) shows the changes in tumor spheroid volume. During different treatments for 5 days, the average volume of the control group increased gradually, while BR2-Lp/CTD at 10 μM CTD concentration treated group decreased in comparison to the other groups. Delayed cytotoxic responses from the BR2-Lp/CTD and Lp/CTD groups at day 1 were observed, possibly resulting from the controlled CTD release profile. Figure 5(D) shows tumor spheroid preservation after treatment such that half of tumor spheroids in BR2-Lp/CTD group were destroyed. Figure 5(E) represents the cell viability data of spheroids treated with different formulations. As shown in this figure, blank liposomes had no apparent cytotoxic effect on the spheroids, consistent with the results in monolayer experiments. This data taken together indicates that free CTD and Lp/CTD have a weak toxic effect on HepG2 spheroids at 10 μM, but that BR2-Lp/CTD has a much stronger effect not only at the end of Day 5 but also earlier. Viability values of spheroids treated with Lp/CTD and BR2-Lp/CTD at 10 μM of CTD concentration were 82.4 ± 7.7 and 73.8 ± 7.4%, respectively, which was significantly lower than the viability values of free CTD and blank Lp groups (*p* < .01). This reduced cell viability in Lp/CTD and BR2-Lp/CTD groups suggests that liposome encapsulation enhanced the cellular uptake and penetration into the spheroids of the drug.

### *In vivo* targeting efficiency and biodistribution

**Figure 6.**
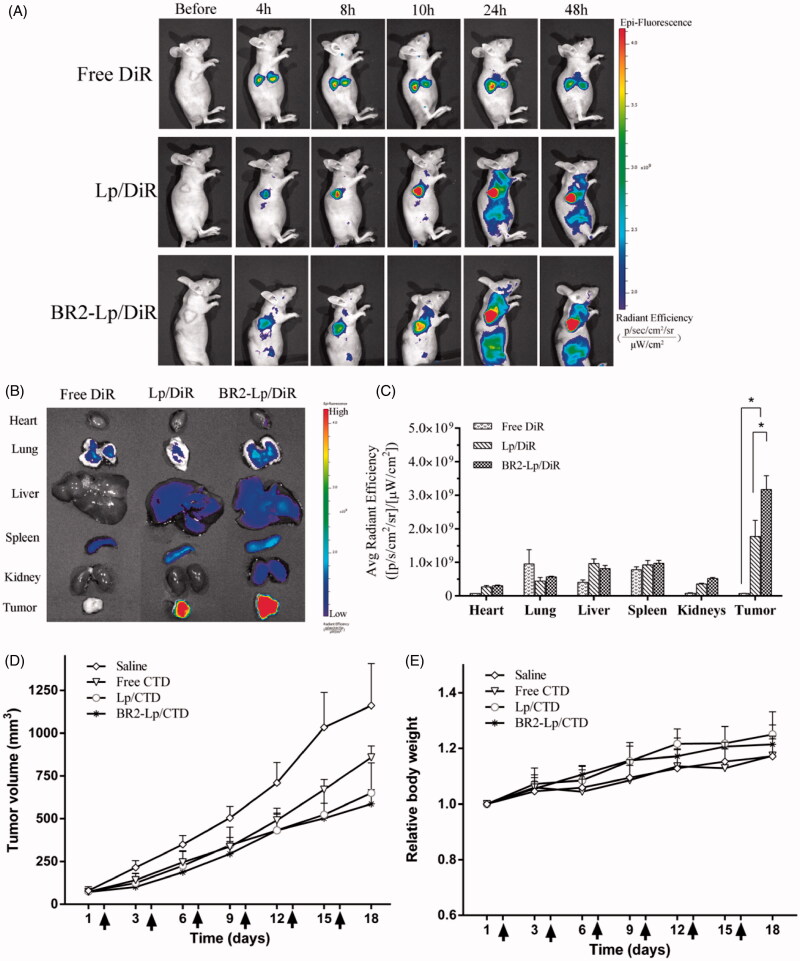
BR2 conjugation facilitates liposomes accumulation and enhances the anticancer efficacy in subcutaneous HepG2 xenograft tumor in nude mice. (A) *In vivo* fluorescence images of HepG2 tumor (150–200 mm^3^) bearing mice at different time points after single intravenous injection of different DiR formulations at dose of 2.0 mg/kg. At 4, 8, 10, 24 and 48 h after injection, mice with *in vivo* DiR fluorescence were imaged by IVIS Spectrum system. (B) After 48-h injection, the mice were sacrificed, and the tumors and vital organs were harvested and placed on a black paper for *ex vivo* imaging. (C) Average fluorescence signals of *ex vivo* tumors and organs after DiR treatment for 48 h *p < .05. (D) Anticancer effect on HepG2-tumor xenograft treated with BR2-Lp/DiR. Tumor volume changes after intravenous injection of saline, free CTD, Lp/CTD and BR2-Lp/CTD at a dose of CTD 0.35 mg/kg for a total of six injections with 3 days interval. (E) Body weight changes profile of tumor-bearing mice after treatment. Arrows indicate the drug administration time.

Figure 6(A) showed the *in vivo* real-time distribution and tumor accumulation ability of DiR loaded liposomes in HepG2 tumor bearing mice. The fluorescence accumulation was perspicuously found in tumors of liposomal DiR treated groups since 4 h post injection, and the signals became much stronger from 8 to 48 h. In contrast, the fluorescence of free DiR treated mice was invisible at the tumor site. As for BR2-Lp/DiR, the fluorescence displayed lower than Lp/DiR group in the first 10 h, but it displayed a sustained and elevated intensity thereafter. Apart from the tumor sites, liposomes were also distributed in the reticuloendothelial system (spleen, liver, lung). But more importantly, the whole-body distribution of BR2-Lp/DiR was reduced subsequently compared to Lp/DiR groups, indicating that BR2-Lp/DiR moved to target tumor sites hence reduced nonspecific distribution. *Ex vivo* images of harvested organs (Figure 6(B)) also confirmed that BR2 conjugation led to much more accumulation of liposomes in tumor sites, and showed a significant difference of BR2-modified liposomes among the groups (*p* < .05) (Figure 6(C)).

### *In vivo* anticancer efficacy

The *in vivo* anticancer efficacy of BR2-modified liposomes encapsulated with CTD was evaluated in HepG2 tumor bearing nude mice in comparison with saline injection as the control. As shown in Figure 6(D), the saline groups displayed a faster tumor growth over the whole period of experiment. Meanwhile, groups treated with free CTD, Lp/CTD and BR2-Lp/CTD showed tumor growth inhibition compared to the control group. Moreover, the tumor volume in BR2-Lp/CTD was smaller than that of free CTD treated mice. As an indicator for the systemic toxicity, the body weight of mice was similar in all groups (Figure 6(E)), indicating that the drug modification did no increase the toxicity to the body. Altogether, the *in vivo* results demonstrate that the modified drug preparation has a stronger anticancer effect without increasing toxicity to the body.

## Discussion

This is the first report of studies demonstrating that the BR2 peptide can be incorporated into nanocarrier liposome for cancer treatment. The study mainly focused on hepatocellular cancer as previous studies have already demonstrated similar effects in other cancer cell lines (Lee et al., 2008; Lim et al., 2013) other than liver cancer that however remains clinically challenging. The results in our study showed an improved BR2-Lp/CTD cytotoxicity on liver cancerous cells compared to unmodified liposomes. BR2-Lp had a pronounced cytotoxic effect on HepG2 cells, as displayed both in MTT assays and images of cellular morphology. Associated cellular studies further verified the specific efficacy of BR2-Lp’s ability to penetrate HepG2 cells. This BR2-functionalized liposome showed higher cytotoxicity and enhanced internalization with regard to HepG2 cells as compared to normal Miha cells. This can be attributed to different cellular membrane structures in the two types of cells, as mentioned before (Lim et al., 2013). It could also be due to the ability of the BR2 peptide to bind specifically to gangliosides, which were increasingly expressed in the tumor tissues as compared to normal tissues (Marquina et al., 1996; Ye et al., 1990).

Based on a recent report of the tumor-specific uptake of BR2 (Lim et al., 2013), we used this peptide as a targeting moiety to facilitate delivery of CTD liposomes in order to obtain selectively target to tumor cells in HCC monolayer and tumor spheroid model. BR2 is known for its efficient ability to penetrate cancer cells based on its specific electrostatic interaction with negatively charged gangliosides on the cancer cell plasma membrane (Lee et al., 2008; Lim et al., 2013). As gangliosides are overexpressed in HCC but little expressed in normal hepatocytes (Tanno et al., 1993), conjugating the BR2 peptide to the liposomal surface could be expected to promote penetration of the drug into HCC cancer cells.

It has been reported that CPP-modified liposomal delivery systems enter cells mostly via endocytosis, subsequently delivering transported cargo to the endosomes or lysosomes (Liang et al., 2015), but these endosomes/lysosomes may then degrade (Maitani and Hattori, 2009). This degradation results in a lower therapeutic potential (Deshpande et al., 2013) as the site of CTD action then becomes cellular nuclei rather than lysosomes (Rauh et al., 2007). By using the LysoTracker probe to track acidic organelles like lysosomes, our study revealed that most of the BR2-Lp that entered cells was not colocalized with lysosomes. Bypassing conventional endocytosis in order to escape lysosomal degradation is beneficial for intracellular drug delivery. These results clearly show that BR2-Lp can be an effective and long-lasting drug delivery vehicle.

As the positive results of monolayer cell culturing might not be enough to confirm tumor penetrating capability as drugs circulating in the blood have difficulty penetrating most solid tumor masses (Kang et al., 2015), tumor spheroids have been proposed as favorable yet simple models for evaluation of targeted delivery and tumor chemotherapy *in vivo* (Mehta et al., 2012). Multicellular tumor spheroids resemble solid tumors in terms of the structural and microenvironmental heterogeneity; therefore, the behavior of nanocarrier in spheroids is likely to reflect its behavior in real tumors (Mikhail et al., 2014; Zhang et al., 2016b). Hence, in our present study, we developed a HepG2 tumor spheroid model to verify the enhanced penetration efficacy and tumor growth inhibition of BR2-Lp on tumor spheroids. In this work, we found that BR2-Lp penetrated roughly 1.4-fold deeper than unmodified liposomes into tumor spheroids. This result was shown by greater mean fluorescent intensity for the BR2-Lp group at different slices as compared to the unmodified liposome group (Figure 4).

In this study, the ability of BR2-Lp/CTD to inhibit the growth of HepG2 tumor spheroids was seen most efficiently in images showing tumor volume changes (Figure 5). The improved efficacy of BR2-Lp might result from the enhanced and more specific penetration and sustained release of CTD molecules. The improved efficacy of BR2-Lp in hindering the proliferation of HepG2 monolayer cells might be due to the same reasons.

*In vivo* biodistribution results in our study further demonstrated the selective accumulation of BR2-Lp in the cancer. This could be attributable to, firstly, liposomes being PEGylated that facilitates the long-term circulation in blood compared to free DiR and therefore more chance to access to target tumor. Secondly, both liposomes with a particle size around 100 nm are in favor of accumulating into tumor sites based on EPR effects in a time-dependent manner. Moreover, BR2 cancer cell peptides are most likely in favor of the liposomes to penetrate and accumulate in the tumor. Furthermore, the enhanced tumor cell-penetrating ability of BR2 could also be related to the lipid-mediated macropinocytosis of interaction with gangliosides that is overexpressed in HCC but little expressed in normal hepatocytes (Tanno et al., 1993; Lee et al., 2008; Lim et al., 2013).

The biodistribution of Lp and BR2-Lp results indicate that both passive and active tumor-targeting mechanisms were involved in DiR accumulation within tumors, where the passive targeting is most likely attributable to the EPR effect of liposomes and active targeting achieved by BR2 conjugation to liposomes. As presented in Figure 6(C), the accumulation of DiR in tumors was considerably increased after the attachment of BR2 onto liposome surface. This evidence suggested that BR2 modification could facilitate the accumulation of liposomes in tumor sites.

The further *in vivo* anticancer efficacy was performed on the subcutaneous tumor bearing mice, and the results showed a lower tumor volume of BR2-Lp/CTD than the other three groups (Figure 6(D)), indicating the enhanced anticancer effects of the preparation when CTD is encapsulated into BR2-modified liposomes.

## Conclusion

In this study, we improved the tumor-selectivity and penetration of liposomes equipped with BR2 for anticancer drug delivery. The *in vitro* and *in vivo* studies both demonstrated that the BR2-modified liposomes delivery system is a preferable and feasible option for anticancer therapy. In particular, our results *in vitro* as well as *in vivo* clearly demonstrate that (1) BR2-modified liposomes loaded with CTD work as a drug carrier and display specific and better anticancer activity against HepG2 cells, and (2) the modified liposomes loaded with CTD have superior penetration, enhanced cellular internalization and improved cytotoxicity against HepG2 tumor spheroids and xenograft tumor.
